# Mechanism of Targeting the Hedgehog Signaling Pathway against Chemotherapeutic Resistance in Multiple Myeloma

**DOI:** 10.1155/2022/1399697

**Published:** 2022-06-29

**Authors:** Yuefeng Zhang, Guoli Yao, Xinxin Yang, Tao Qiu, Sheng Wang

**Affiliations:** Department of Hematology, First People's Hospital of Linping District, Hangzhou 311100, Zhejiang, China

## Abstract

**Objective:**

The aim of this study was to explore the relationship between the Hedgehog signaling pathway and drug resistance in multiple myeloma.

**Methods:**

The human myeloma cell line RPMI 8266 was taken as the research object. An azithromycin (AZM)-resistant cell line RPMI 8226/R was constructed, and GENT61 was used to block the Hedgehog signaling pathway. Cells were rolled into RPMI 8226/S (S group), RPMI 8226/R (R group), GENT61+RPMI 8226/S (GENT61+S group), and GENT61+RPMI 8226/R (GENT61+R group). The proliferation of cells in each group was assessed, and the expression of patched homolog 1 (PTCH1), zinc finger-containing transcription factors 1 (GLI1), GLI2, hair-division associated enhancer 1 (Hes1), and sonic hedgehog factor (SHH) in each group was detected. Interleukin (IL)-6 and vascular endothelial growth factor (VEGF) were measured.

**Results:**

Compared with the S group, the expression levels of PTCH1, GLI2, Hes1, and SHH and the contents of IL-6 and VEGF in the *R* group were greatly increased, while the expression level of GLI1 was notably decreased (*P* < 0.05). Compared with the *R* group, the GENT61+R group greatly increased cell proliferation inhibition. However, the expression levels of PTCH1, GLI2, Hes1, and SHH, and the contents of IL-6 and VEGF were notably decreased, while GLI1 expression levels were greatly increased (*P* < 0.05).

**Conclusion:**

AZM-resistant multiple myeloma was closely associated with the Hedgehog signaling pathway activation, and blocking the Hedgehog signaling pathway can be used as a therapeutic target to improve drug resistance in multiple myeloma.

## 1. Introduction

Multiple myeloma (MM) is a malignant plasma cell disease characterized by abnormal proliferation of bone marrow plasma cells with excessive growth of monoclonal immunoglobulin or light chain (M protein), which inhibits the normal function of immunoglobulin after excessive production of monoclonal immunoglobulin, resulting in the involvement of various organs in the body [1]. The main clinical features of multiple myeloma patients are bone destruction, incomplete renal function, repeated infections, etc. [2]. Clinical statistics showed that multiple myeloma is more common in middle-aged and elderly groups, which has become the second largest hematological tumor disease [3]. Hematopoietic stem cell transplantation can effectively cure multiple myeloma, but it is characterized by a low cure rate, large side effects, high treatment cost, and high mortality, so it cannot be effectively promoted and applied in clinical practice [4]. Currently, chemotherapy/radiotherapy is mostly used for the treatment of multiple myeloma. However, there are still some disadvantages such as high recurrence rate and large side effects after treatment [5]. The main factor in the failure of chemotherapy for multiple myeloma is drug resistance [6]. Therefore, it is very important to find therapeutic targets that can effectively reverse drug resistance of chemotherapy.

The mechanism of drug resistance in multiple myeloma is very complex, which has been confirmed to be related to cytogenetics, bone marrow microenvironment, and signaling pathway transmission [7]. Myeloma and its microenvironment often contain abundant cytokines, such as interleukin (IL)-6, vascular endothelial growth factor (VEGF), and insulin-like growth factor (IGF), which promote the proliferation of myeloma cells [8]. The survival, proliferation, and drug resistance of multiple myeloma cells are related to a variety of signaling pathways, such as the Wnt signaling pathway, Notch signaling pathway, and Hedgehog signaling pathway [9–11]. Studies have confirmed that the Hedgehog signaling pathway is involved in embryo development as well as tissue and organ formation, which also mediates the occurrence and progression of tumors [12]. Hedgehog signaling is a ligand-dependent pathway, including sonic hedgehog (SHH), Indian hedgehog (Ihh), and desert hedgehog (Dhh) ligands, PTCH1 or PTCH2, binding SMO of PTCH in inactive state, and downstream transcription factors GIL1, GLI2, and GLI3 [13].

However, there is relatively little evidence on whether the Hedgehog signaling pathway can participate in and regulate the process and drug resistance of multiple myeloma. Therefore, to explore the mechanism of the Hedgehog signaling pathway on resistance to multiple myeloma, the human myeloma cell line RPMI 8226 was used to construct the azithromycin (AZM)-resistant cell line RPMI 8226/R. The effects of Hedgehog signaling pathway blocker GANT61 on proliferation, IL-6, and VEGF cytokine levels of drug-resistant cells were compared. This research was developed to understand the correlation between the Hedgehog signaling pathway and drug resistance in multiple myeloma patients and to provide experimental data for improving the therapeutic effect of multiple myeloma chemotherapy patients.

## 2. Materials and Methods

### 2.1. Experimental Materials

The human myeloma cell line RPMI 8226 was purchased from Shanghai Tongpai Biotechnology Co., Ltd. Fetal bovine serum and RPMI 1640 medium were purchased from Gibco, USA. AZM and GANT61 were purchased from AbMole, USA. The Trizol reagent, RIPA lysate, and enzyme-linked immunosorbent assay (ELISA) kit were purchased from Invitrogen, USA. PrimeScript™ RT reagent Kit (Perfect Real Time) and TB Green® Premix Ex Taq™ (Tli RNaseH Plus) Bulk were purchased from Takara, Japan. Cell counting kit-8 (CKK-8) detection kit, rabbit polyclonal PTCH1 primary antibody, rabbit monoclonal GLI1 primary antibody, mouse GLI2 monoclonal antibody, rabbit Hes1 monoclonal antibody, rabbit Sonic Hedgehog monoclonal antibody, rabbit polyclonal beta-actin primary antibody, and horseradish peroxidase-labeled rabbit anti-human IgG secondary antibody were purchased from Abcam. The polyvinylidene fluoride (PVDF) membrane, bicinchoninic acid (BCA) kit, and electrochemiluminescence (ECL) chemiluminescence detection kit were purchased from Shanghai Beyotime Biotechnology Co., LTD.

### 2.2. Construction of AZM-Sensitive and AZM-Resistant Cell Lines

The AZM-sensitive cell line RPMI8226/S was constructed by using RPMI1640 medium containing 13% fetal bovine serum in a cell incubator containing 5% CO_2_ at 37°C for 48 h and adding AZM medium with a 0.1 *μ*M final concentration. Cells were exchanged at 48-hour intervals, and the AZM concentration increased gradually. The culture lasted for 10 months. When the final AZM concentration reached 2.0 *μ*M, the increase in AZM concentration stopped, and the resistant cell line RPMI8226/R was cultured in a normal cell medium.

### 2.3. Grouping of Cells

The AZM-sensitive cell line RPMI 8226/S and AZM-resistant cell line RPMI 8226/R in the logarithmic growth stage were taken, and the cells were inoculated into 96-well plates at a concentration of 1 × 10^3^ cells/well. Cells were cultured using RPMI 1640 cell medium containing 13% fetal bovine serum. The cells were rolled into four groups based on cell culture methods: Group S: RPMI8228/S cells were cultured normally; group R: RPMI8228/R cells were cultured normally; GANT61+S group: RPMI8228/S cells were cultured normally and treated with GANT61 at 20 *μ*mol/L; GANT61+R group: RPMI8228/R cells were cultured normally and treated with GANT61 at 20 *μ*mol/L.

### 2.4. Cell Proliferation Detected by CCK-8

Cells in each group with good growth status were taken and centrifuged at 1,000 rpm for 5 min, then the medium was discarded, and a cell suspension was prepared using the new medium. The cells were seeded in 96-well plates, 10 *μ*L CCK-8 reagent was added to each well after cell adherence, and the culture was continued for 2 h. The well without reagent and cells was set as the blank group, and the well containing reagent only was set as the control well. The absorbance (OD) of cells in each well was measured at 450 nm using a multifunctional microplate reader. Then, the cell proliferation inhibition rate was calculated according to the equation ((OD _control group_ − OD _experimental group_)/(OD _control group_ − OD _blank group_)) × 100%.

### 2.5. Real-Time Fluorescence Quantitative PCR Detection

After grouping and corresponding treatments, cells in each group were collected and centrifuged at 1,000 rpm for 5 min before the culture medium was discarded. Total RNA was extracted from cells according to the Trizol method. A nucleic acid detector was used to detect the concentration and purity of extracted RNA, and a 2% agarose gel electrophoresis was used to detect the integrity of extracted RNA. The extracted RNA was used as a template, followed by reverse transcription of cDNA according to the PrimeScript™ RT Reagent Kit (Perfect Real Time) instructions. Using cDNA obtained by reverse transcription as a template, the reaction system, and reaction procedure were set according to the TB Green® Premix Ex Taq™ (Tli RNaseH Plus), Bulk kit instructions. The total reaction system was set as follows: 2.0 *μ*L cDNA template, 12.5 *μ*L TB Green Premix Ex Taq (2×), 0.5 *μ*L upstream PCR primer, 0.5 *μ*L downstream PCR primer, and 9.5 *μ*L ddH_2_O. The total reaction system was 25 *μ*L. The reaction amplification program was set as 95°C 30 s, 95°C 5 s⟶60°C 30 s (40 cycles), and 72°C 30 s. All the quantitative primers used in the study were designed and synthesized by Shanghai Sangon Bioengineering Co., Ltd. The primer information is shown in Table 1. Then, the Ct value was read. Accordingly, equations ΔCt = Ct _target gene_ − Ct _reference gene_ and ΔΔCt = ΔCt _experimental group_ − ΔCt _control group_, 2^−ΔΔCt^ were used to test the relative expression level of the target gene.

### 2.6. Western Blotting

After the grouping, cells in each group were collected and centrifuged at 1,000 rpm for 5 min before the culture medium was discarded. After the cells were washed with precooled phosphoric acid buffer, an appropriate amount of cell lysis solution was added for cell lysis. After centrifugation at 12,000 rpm for 20 min, the supernatant was taken, and the concentration of extracted protein was quantitatively determined according to the BCA kit instructions. The extracted sample protein was placed in a constant temperature metal bath at 98°C for 10 min to denaturize the protein, and the corresponding concentrations of separation glue and concentrated glue were configured. After the sample protein was sampled, electrophoresis was performed. After protein separation, the glue was cut and the protein was transferred to a PVDF membrane for 2 h. The PVDF membrane was placed in a sealed solution containing 5% skim milk powder and shaken at room temperature for 2 h. Then, the primary antibodies diluted with blocking solution, PTCH1 (1 : 1,000), GLI1 (1 : 1,000), GLI2 (1 : 500), Hes1 (1 : 1,000), SHH (1 : 10,000), and *β*-actin (1 : 5,000) were added, shaken well, and incubated overnight in a refrigerator at 4°C. The PVDF membranes were rinsed with 1 × Tris-buffered saline Tween (TBST) three times, and the horseradish peroxidase-labeled IgG (1 : 25,000) secondary antibody diluted with block solution was added. After shaking, the PVDF membranes were incubated at room temperature for 2 h. The PVDF membrane was rinsed with 1 × TBST three times, and the target protein bands were developed according to the ECL chemiluminescence detection kit. Photographs were taken using a gel imager and quantitative analysis of the bands of the target protein was performed using a Gel-Pro Analyzer. Using *β*-actin as a reference gene, the relative expression level of the target gene protein was calculated.

### 2.7. ELISA

The cells were cultured on 24-well plates and starved in a serum-free cell culture medium for 24 h before cell grouping. Further culture and centrifugation were performed to obtain the supernatant. According to the ELISA kit instructions, IL-6 and VEGF content in cell fluid were detected. Pure diluent was added and used as a blank well, and standard was added and used as a standard well. A 100 *μ*L solution was added to each well of the ELISA plate and incubated for 1.5 h at 36°C. Then, 350 *μ*L washing solution was added to each well. After the wells were dry, 100 *μ*L biotinylated antibody working solution was added to each well and incubated at 36°C for 1 h. Then, 100 *μ*L enzyme binding solution was added and incubated at 36°C for 30 min under dark conditions. The plate was washed and added with 100 *μ*L color developer solution, incubated at 36°C for 15 min away from light. Finally, 100 *μ*L reaction stop solution was added to each well, and the absorbance of cells in each well was measured at 450 nm with a microplate reader.

### 2.8. Observation Indexes

- Real-time quantitative PCR was used to detect the mRNA expression levels of target genes in the hedgehog signaling pathway in RPMI8226/S and RPMI8226/R cell lines
- Western blotting was used to detect the protein expression levels of target genes in the Hedgehog signaling pathway in RPMI8226/S and RPMI8226/R cell lines
- After treatment with GANT61 blocker, the mRNA expression levels of target genes of the Hedgehog signaling pathway in RPMI8226/S and RPMI8226/R cells were detected by real-time fluorescence quantitative PCR
- Western blot was used to detect the protein expression levels of target genes of the Hedgehog signaling pathway in RPMI8226/S and RPMI8226/R cells treated with GANT61 blocker
- The proliferation inhibition of myeloma cells in each group was detected by the CCK-8 assay
- The content of IL-6 and VEGF in cell supernatant was detected by ELISA

### 2.9. Statistical Processing

The SPSS 19.0 was employed for collation and statistical analysis of experimental data. All experimental data were expressed as mean ± standard deviation, and one-way ANOVA was used for statistical comparison of differences between the groups. When *P* < 0.05, the difference was statistically significant.

## 3. Results

### 3.1. Comparison of Hedgehog Pathway Activity between Group S and Group R

**Figure 1:**
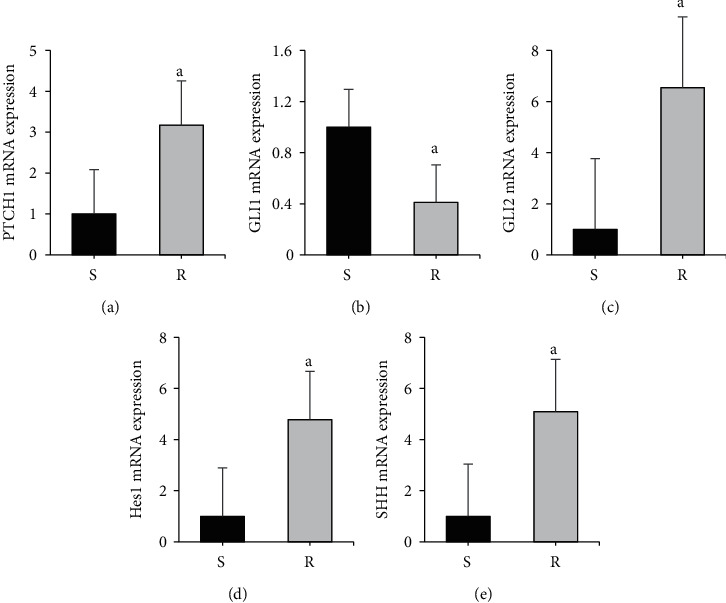
Comparison of mRNA expression differences of target genes in AZM-sensitive and drug-resistant myeloma cells. Note: A was PTCH1 level; B was GLI1 level; C was GLI2 level; D was Hes1 level; E was SHH level; compared with the S group, ^*a*^*P* < 0.05.

Figure 1 shows the comparison of mRNA expression differences of target genes in AZM-sensitive and drug-resistant myeloma cells. The RNA expression levels of PTCH1, GLI2, Hes1, and SHH in the *R* group were considerably superior to those in the S group, with statistical significance (*P* < 0.05). The GLI1 mRNA expression level in the R group was dramatically inferior to that in the S group (*P* < 0.05).

**Figure 2:**
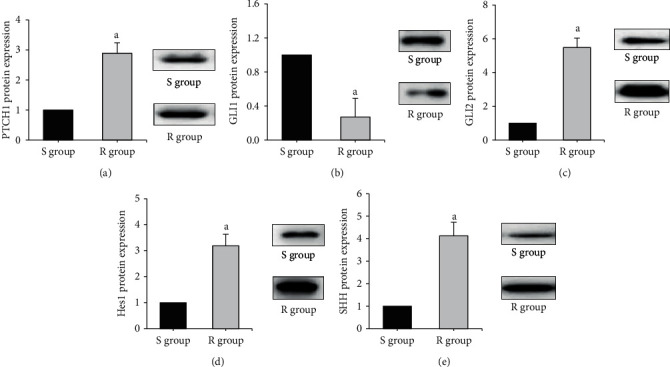
Comparison of target gene protein expression in AZM-sensitive and drug-resistant myeloma cells. (Note: A is PTCH1; B is GLI1; C is GLI2; D is Hes1; E is SHH, and a means statistically different from group S (*P* < 0.05)).

Figure 2 shows the comparison of target gene protein expression in AZM-sensitive and drug-resistant myeloma cells. The protein expression levels of PTCH1, GLI2, Hes1, and SHH in the R group were considerably superior to those in the S group, with statistical significance (*P* < 0.05). The GLI1 protein expression level in the *R* group was dramatically inferior to that in the S group (*P* < 0.05).

### 3.2. Effects of GANT61 Blocker on the Hedgehog Pathway in Drug-Resistant Myeloma Cells

**Figure 3:**
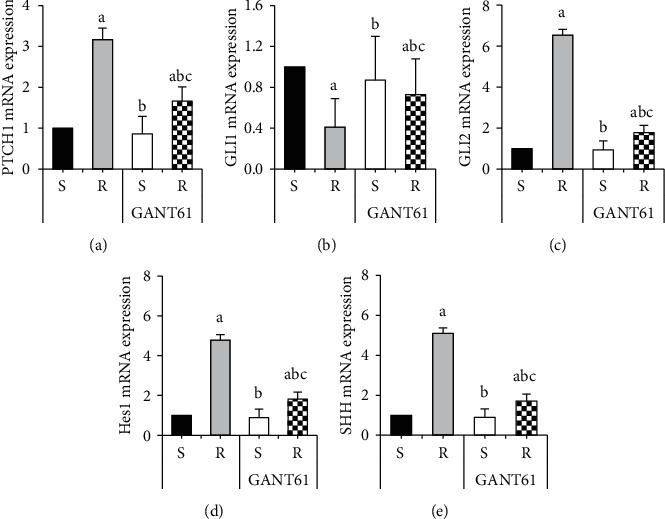
Comparison of mRNA expression differences of target genes in AZM-sensitive and drug-resistant myeloma cells treated with GANT61. Note: A was PTCH1 level; B was GLI1 level; C was GLI2 level; D was Hes1 level; E was SHH level; compared with the S group, ^*a*^*P* < 0.05; compared with the R group, ^*b*^*P* < 0.05; compared with the GANT61+S group, ^*c*^*P* < 0.05.

Changes in mRNA expression levels of target genes of the Hedgehog signaling pathway in RPMI 8226/S and RPMI 8226/R cells after treatment with GANT61 blocker were detected by real-time fluorescence quantitative PCR (Figure 3). After treatment with GANT61 blocker, the mRNA expression levels of PTCH1, GLI2, Hes1, and SHH in the GANT61+S group and GANT61+R groups were dramatically inferior to those in the *R* group (*P* < 0.05). The GLI1 mRNA expression level in the GANT61+S group and the GANT61+R group was considerably superior to that in the *R* group (*P* < 0.05. However, the mRNA expression levels of PTCH1, GLI2, Hes1, and SHH in the GANT61+R group were considerably superior to those in the GANT61+S group, with statistical significance (*P* < 0.05). The GLI1 mRNA expression level in the GANT61+R group was dramatically inferior to that in the GANT61+S group (*P* < 0.05).

**Figure 4:**
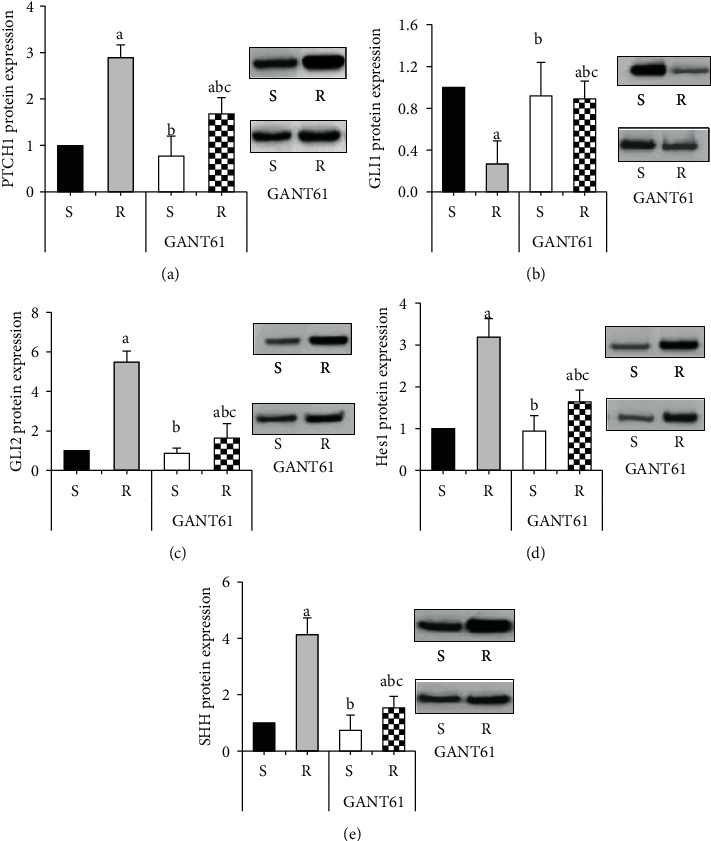
Comparison of target gene protein expression in AZM-sensitive and drug-resistant myeloma cells treated with GANT61. (Note: A is PTCH1; B is GLI1; C is GLI2; *D* is Hes1; *E* is SHH, and a means statistically different from group S (*P* < 0.05), *b* represents a statistical difference compared with the *R* group (*P* < 0.05), *c* represents a statistical difference compared with the GANT61+S group (*P* < 0.05).

A western blot was used to detect the changes in protein expression levels of target genes of the Hedgehog signaling pathway in RPMI 8226/S and RPMI 8226/R cells after treatment with GANT61 blocker (Figure 4). After treatment with GANT61 blocker, the protein expression levels of PTCH1, GLI2, Hes1, and SHH in the GANT61+S and GANT61+R groups were dramatically inferior to those in the *R* group, with statistical significance (*P* < 0.05). The GLI1 protein expression level in the GANT61+S group and the GANT61+R group was considerably superior to that in the *R* group (*P* < 0.05). However, the protein expression levels of PTCH1, GLI2, Hes1, and SHH in the GANT61+R group were considerably superior to those in the GANT61+S group, with statistical significance (*P* < 0.05). The GLI1 protein expression level in the GANT61+R group was dramatically inferior to that in the GANT61+S group (*P* < 0.05).

### 3.3. Effect of Blocking the Hedgehog Pathway on Proliferation Inhibition of Drug-Resistant Myeloma Cells

**Figure 5:**
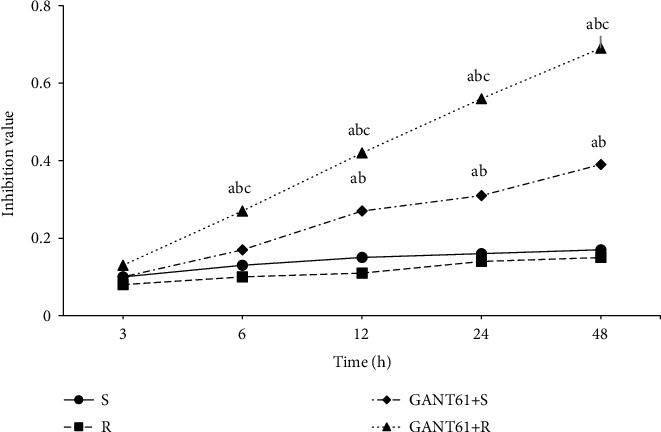
Proliferation inhibition curve of myeloma cells of different culture time. Note: compared with the S group, ^*a*^*P* < 0.05; compared with the R group, ^*b*^*P* < 0.05; compared with the GANT61+S group, ^*c*^*P* < 0.05.

Figure 5 shows the proliferation inhibition curves of myeloma cells at different culture times. With the extension of culture time, the proliferation inhibition of cells in the S group and *R* group did not change remarkably, and there was no statistical significance between the groups (*P* > 0.05). The proliferation inhibition of the GANT61+S group and GANT61+R group increased with the extension of culture time, and the proliferation inhibition was considerably superior to that of the S group and *R* group (*P* < 0.05). The inhibitory effect of the GANT61+R group was considerably superior to that of the GANT61+S group (*P* < 0.05).

### 3.4. Effects of Blocking the Hedgehog Pathway on IL-6 and VEGF Levels in Myeloma Drug-Resistant Cells

**Figure 6:**
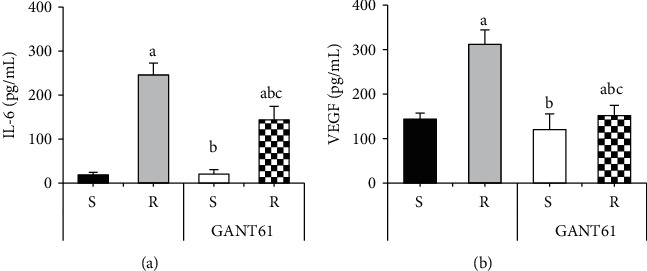
Comparison of IL-6 and VEGF contents in AZM-sensitive and drug-resistant myeloma cells treated with GANT61. Note: A was IL-6 content; B was VEGF content; compared with the S group, ^*a*^*P* < 0.05; compared with the R group, ^*b*^*P* < 0.05; compared with the GANT61+S group, ^*c*^*P* < 0.05.

Figure 6 shows the comparison of IL-6 and VEGF levels in AZM-sensitive and drug-resistant myeloma cells treated with GANT61. Figure 6 showed that the contents of IL-6 and VEGF in the *R* group were considerably superior to those in the S group (*P* < 0.05). After treatment with GANT61 blocker, the expression levels of IL-6 and VEGF in GANT61+S group and GANT61+R group were dramatically inferior to those in the *R* group, with statistical significance (*P* < 0.05). The levels of IL-6 and VEGF in the GANT61+R group were considerably superior to those in the GANT61+S group, with statistical significance (*P* < 0.05).

## 4. Discussion

Multiple myeloma is mostly treated with drugs or hematopoietic stem cell transplantation [14, 15]. Currently, new molecular-targeted drugs and hematopoietic stem cell transplantation have been used in the clinical treatment of multiple myeloma, which can significantly improve the clinical symptoms and prognosis of patients, but the recurrence rate is still very high [16, 17]. The Hedgehog signaling pathway is involved in biological processes such as embryonic development, cell differentiation, and cell proliferation, and also participates in and mediates multiple malignant tumor diseases, such as multiple myeloma, glioma, and medulloblastoma, playing an important role in the survival and proliferation of multiple myeloma cells [18, 19].

The Hedgehog signaling pathway mainly includes three ligands, namely SHH, IHH, and DHH. Of those, SHH is mainly expressed in the nervous system and other tissues [20]. However, there are two common receptors in the Hedgehog signaling pathway, PTCH1 and PTCH2. PTCH without ligand can bind to SMO protein and promote the expression of transcription factor GLI [21]. Therefore, the target genes of the Hedgehog signaling pathway include PTCH1, PTCH2, GLI1, and GLI2 [22]. Hes1 is one of the target genes of the Hedgehog signaling pathway, which is involved in regulating cell differentiation, proliferation, intracellular gene transcription, etc. [23]. In this study, the expression changes of PTCH1, GLI1, GLI2, Hes1, and SHH in the multiple myeloma drug-resistant cell line RPMI 8226/R were first detected. The results showed that the expression levels of PTCH1, GLI2, Hes1, and SHH in RPMI 8226/R were greatly increased compared with the multiple myeloma drug-sensitive cell line RPMI 8226/S, while the expression level of GLI1 was notably decreased. In addition, compared with RPMI 8226/S, the levels of IL-6 and VEGF in RPMI 8226/R were greatly increased. Multiple myeloma cells can bind to cellulose in the microenvironment and generate cell adhesion-mediated chemotherapeutic drug resistance. However, cytokines such as IL-6 and VEGF can induce the activation of a variety of signaling pathways and promote cell adhesion-mediated drug resistance, thus promoting the proliferation of multiple myeloma cells and inhibiting cell apoptosis [24, 25].

Currently, known inhibitors of the Hedgehog signaling pathway include GANT61, cyclopamine, and GANT58 [26]. GANT61 specifically blocks GLI1 in the Hedgehog signaling pathway and inhibits cell transcription and expression [27]. Multiple myeloma cells were treated with GANT61 as a Hedgehog signaling pathway blocker. The results showed that the proliferation inhibition of RPMI 8226/R cells increased remarkably after GANT61 treatment. However, the expression levels of PTCH1, GLI2, Hes1, and SHH in the cells were notably decreased, and GLI1 expression levels were greatly increased. These results indicate that GANT61 can effectively inhibit the abnormal activation of the Hedgehog signaling pathway in RPMI 8226/R cells and inhibit the proliferation of RPMI 8226/R cells [28]. Subsequently, IL-6 and VEGF levels in RPMI 8226/R cells were notably decreased after GANT61 treatment. Hence, blocking abnormal activation of the Hedgehog signaling pathway can improve the inflammatory response in the tumor microenvironment and then reverse drug resistance in multiple myeloma.

## 5. Conclusion

In this study, the effect of Hedgehog signaling pathway blocker GANT61 on cell drug resistance was investigated. The results showed that AZM-resistant multiple myeloma was closely related to the activation of the Hedgehog signaling pathway. RPMI8226/R cells blocked the Hedgehog signaling pathway to inhibit the proliferation of RPMI8226/R and reduced the secretion of cytokines such as inflammation, which could be used as a therapeutic target to improve the drug resistance of multiple myeloma. The shortcoming of this study is that only the effects of blocking the Hedgehog signaling pathway on the proliferation and expression of RPMI 8226/R inflammatory factors were analyzed, and the effects of blocking the Hedgehog signaling pathway on other aspects of RPMI 8226/R were not explored. The future research direction is to further explore the mechanism of GANT61 on apoptosis, differentiation, and metastasis of RPMI8226/R cells.

## Figures and Tables

**Table 1:** Quantitative primer information.

Gene	Primer sequences (5′⟶3′)	Product length (bp)
PTCH1	F: GAAGAAGGTGCTAATGTCCTGAC R: GTCCCAGACTGTAATTTCGCC	245

GLI1	F: AGCGTGAGCCTGAATCTGTG R: CAGCATGTACTGGGCTTTGAA	188

GLI2	F: CATGGAGCACTACCTCCGTTC R: CGAGGGTCATCTGGTGGTAAT	173

Hes1	F: TCAACACGACACCGGATAAAC R: GCCGCGAGCTATCTTTCTTCA	153

SHH	F: GAAACTCCGAGCGATTTAAGGA R: GGCCCTCGTAGTGCAGAGA	228

*β*-Actin	F: GGGATTTCGACTTTTGGCTACA R: CTTCCACTACTGCTGTTAGGTG	113

## Data Availability

The data used to support the findings of this study are included within the article.
